# Revisiting the Role of Local Cryotherapy for Acne Treatment: A Review and Update

**DOI:** 10.3390/jcm12010026

**Published:** 2022-12-20

**Authors:** Nark-Kyoung Rho

**Affiliations:** Leaders Aesthetic Laser & Cosmetic Surgery Center, Seoul 06014, Republic of Korea; rhonark@hanmail.net

**Keywords:** acne vulgaris, analgesia, cryosurgery, cryotherapy, hidradenitis suppurativa, intralesional injections, pain, sebaceous gland

## Abstract

Acne vulgaris is a well-recognized condition among adolescents and adults that adversely affects their quality of life. Local cryotherapy has long been reported to be effective in treating acne vulgaris, inducing a more rapid involution of acne than topical medications. However, the use of cryotherapy has been limited for acne treatment due to several drawbacks, including procedural pain and pigmentary alterations. Currently, newer cryotherapy devices are gaining attention in dermatology due to their ability to monitor and precisely control the target temperature. In this narrative review, a brief history and the latest update on acne cryotherapy will be presented. Additionally, a special emphasis is placed on the role of cryotherapy, alone or in combination with intralesional steroid injections for nodulocystic acne.

## 1. Introduction

Since acne vulgaris is a multifactorial disorder, dermatologists often provide different treatment modalities to attack as many factors as possible. Along with topical or systemic medications, physical procedures have been employed for many years to complement medical therapy for acne, from simple extraction to energy-based treatments [1]. Dermatologists use cryotherapy as an inexpensive, safe, and easy way to treat skin lesions in a clinical setting. However, it is still relatively unknown today that local cryotherapy is one of the most frequently used physical modalities to treat acne and has been used for several decades. In this review, we provide a historical perspective and recent updates on the use of cryotherapy to treat acne and related conditions.

## 2. Brief History and Early Reports of Acne Cryotherapy

**Figure 1:**
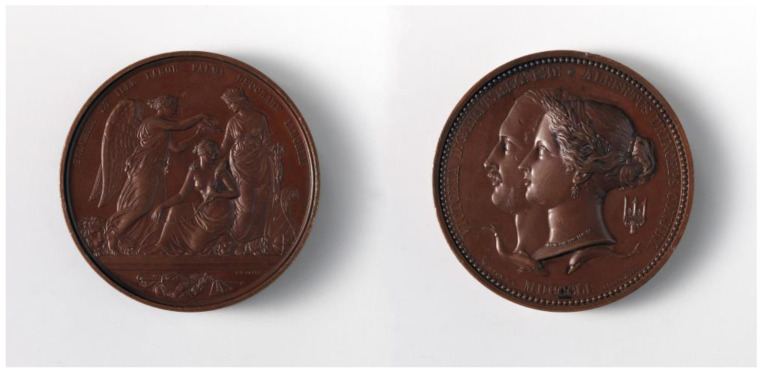
Juror’s medal awarded to Dr. James Arnott, Great Exhibition of London, 1851, for his development of cold therapy equipment (reproduced from the Metropolitan Museum of Art, New York City, used under a Creative Commons Attribution 1.0 Universal Public Domain Dedication).

Dermatologic cryosurgery textbooks and scholarly reviews credit James Arnott, who is widely regarded, as the “father of modern cryosurgery” [2,3]. In his own words, Arnott described his cryotherapy technique as “congelation arresting the accompanying inflammation and destroying the vitality of the cancer cell [2]”. In addition to treating tumors, Arnott also proposed that cryotherapy could be utilized to treat other dermatologic conditions, including acne vulgaris. Arnott won the prize medal at the Great Exhibition of London of 1851 for his cold equipment that allowed reducing tissue temperature to −20 °C [4] (Figure 1).

**Figure 2:**
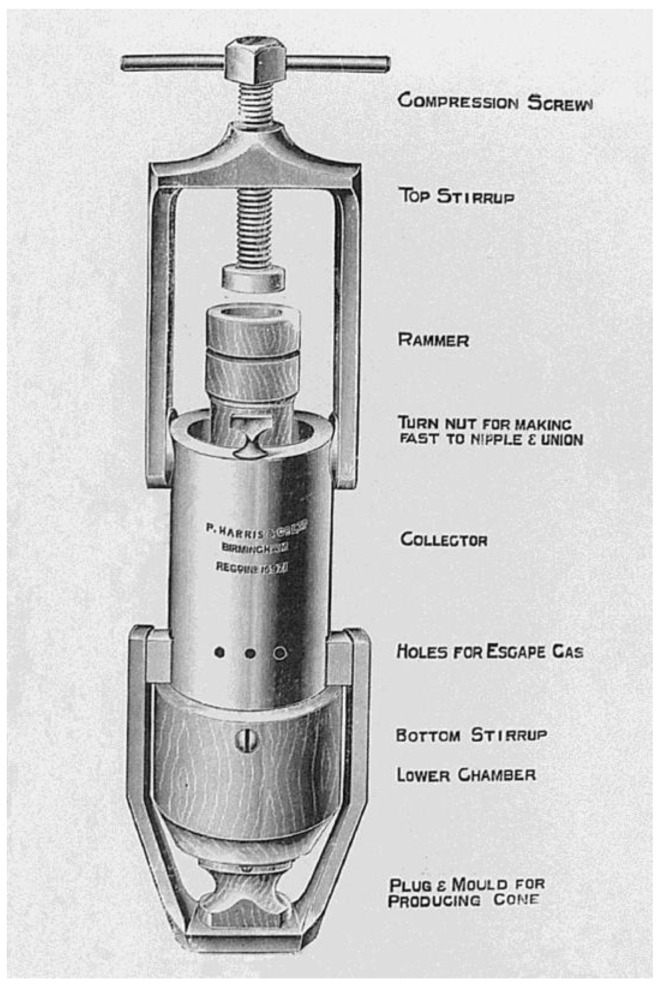
Carbon dioxide snow collector and compressor, as demonstrated by Hall-Edwards 1913 (reproduced from the Wellcome Library, London, used under a Creative Commons Attribution 4.0 International License).

From Arnott’s early work, the practice of cryotherapy has blossomed into a staple in the practice of modern dermatology [5]. Arnott’s idea led to the development of a more practical cryotherapy device consisting of carbon dioxide collector and compressor units, which John Hall-Edwards described in 1911 [6] (Figure 2). In 1925 Giraudeau (cited by [7]) commenced using cryotherapy for acne, with a mixture of solid carbon dioxide (−78.5 °C), acetone, and precipitated sulfur, which was later found out by Dobes et al. [8] to produce better results in papulopustular acne than in nodular lesions. The degree of erythema and desquamation produced by cryotherapy is determined by the time the slush is in contact with the skin [9].

**Figure 3:**
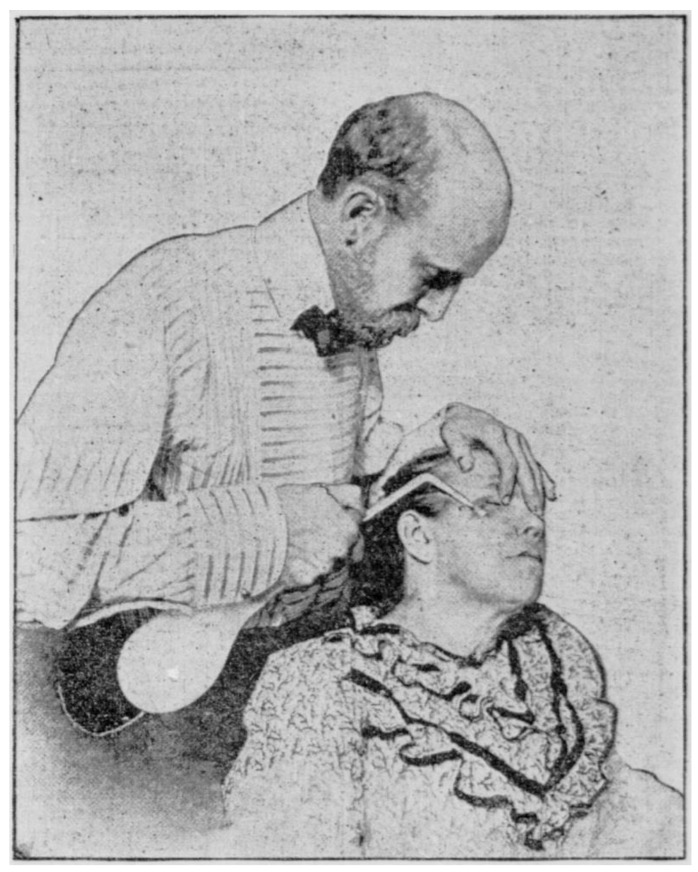
Campbell White treating a skin lesion with sprayed liquid air as it appeared in the New York Tribune in 1900 (reproduced from the Library of Congress, Washington, DC. New York Daily Tribune, October 25, 1900, page 8, image 22. In the public domain).

At the end of the 19th century, all the so-called “permanent gases” (oxygen, nitrogen, and hydrogen) were liquefied, and commercial liquefaction of air was established by Carl Von Linde [4,6]. Campbell White used a glass flask that acted as a liquid air sprayer, which became the first portable cryosurgery device (Figure 3) [4]. During the 1920s and 1930s, liquified oxygen (−182.9 °C) was used as a cryogenic agent to treat various skin conditions, including acne [3]. However, liquid oxygen soon became obsolete as a cryogenic agent because of its high combustibility [10].

Liquid nitrogen (−196 °C) became commercially available and was introduced into clinical practice in 1950 by Herman V. Allington [11], who was the first to publish on the successful use of liquid nitrogen to treat acne. Allington used a cotton swab dipped in liquid nitrogen [4,11]. Later reports in the 1970s mainly used liquid nitrogen as a cryogen source to treat acne. A study of 150 acne patients treated with liquid nitrogen cryotherapy reported excellent results in 95% of cases [12]. In 1973, Goette [13] also reported that liquid nitrogen cryotherapy was effective in treating acne. In 1967, Setrag Zacarian, who brought the term “cryosurgery” into use for the first time, designed a handheld cryosurgical device using liquid nitrogen which gave rise to several models of handheld cryosurgical units [14].

## 3. Cryotherapy for Acne

Key findings from several clinical studies are summarized in Table 1. Prior to the advent of effective medications, cryotherapy was en vogue in the management of acne patients, however, this approach seems to be regarded as obsolete nowadays, as mentioned by Plewig and Kligman [15]. Modern oral and topical antibiotic regimens and oral isotretinoin have reduced the need for cryosurgical treatments of acne [16]. Although cryosurgery is not a first-line modality of current acne therapy, it can be a helpful alternative when treating patients with acne that cannot be treated either with oral or topical medication, for example, acne during pregnancy [16,17].

### 3.1. Cystic Acne

For nodulocystic lesions of acne conglobata, a “cryoprobe” method has been applied with acceptable and long-term results [18]. The superficial freezing with liquid nitrogen fastens the resolution of chronic fluctuant nodular lesions and reduces pain [9]. Based on their experience treating more than 2000 patients with cystic or severe acne vulgaris, Wright and Gross [19] observed that cystic acne lesions almost invariably disappear after a few cryotherapy sessions. In a comparative study Cunliffe [20] confirmed the findings of Graham [12] that liquid nitrogen cryotherapy is preferable to intralesional triamcinolone injection when treating cystic acne. Past clinical experiences suggested that cryotherapy may be most effective in treating superficial cystic lesions and least effective against deeper lesions [13]. One hospital-based study from Nepal shows that cryotherapy is still actively used to treat benign conditions including acne cysts, constituting 4% of dermatology patients [21].

### 3.2. Acne Keloids

Cryotherapy is well described and still frequently used to treat the keloidal type of acne. In 1994, the American Academy of Dermatology’s Committee on guidelines of care stated that cryotherapy is an established treatment for keloids and hypertrophic scars [22]. Zouboulis [23] also suggested that cryotherapy could be the treatment of choice for keloids and hypertrophic scars. Röhrs et al. [24] reported that acne keloids showed a 73% improvement or complete regression after multiple cryotherapy sessions in 16 patients. Resistance to the treatment occurred in 12% of the lesions, mostly observed in cases of larger keloids. Excellent and good results were particularly observed in short-duration cases, whereas older keloids showed poor results. Younger patients showed better responses than older patients. The data from a double-blind study by Layton et al. [25] should also be mentioned. The study compared the efficacy and safety of intralesional triamcinolone and cryosurgery for treating acne keloids. The authors demonstrated that by treating early keloids with the latter, 85% showed a moderate to good response regarding lesion flattening. The response to cryosurgery resulted in a significantly better response in early acne keloids characterized by high vascularity. It is recommended that cryotherapy be performed directly before administering intralesional corticosteroid injections [26] because cryotherapy-induced edema facilitates injection into acne nodules and keloids [27].

### 3.3. Other Subtypes of Acne

**Figure 4:**
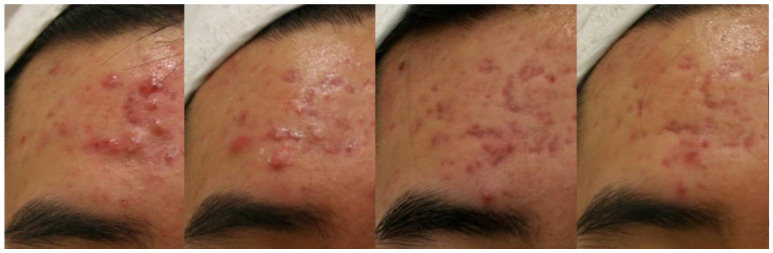
Improvement of inflammatory papules and superficial cysts on the forehead of a Korean adolescent acne patient, after three sessions of local cryotherapy using a temperature-controlled cryotherapy device (TargetCool; RecensMedical, Ulsan, Korea). Permission and consent were given for the use of the photographs.

The efficacy of cryotherapy in comedonal acne is controversial. Earlier researchers [8,13,15] reported that comedonal acne responded poorly to cryotherapy. A split-face controlled trial study of 25 patients in which liquid nitrogen was applied to acne lesions on one side of the face, and other topical therapies were used on the other side, showed liquid nitrogen to be effective against pustular but not comedonal acne [13]. Plewig and Kligman [15] also noted that although spot cryotherapy is moderately helpful in treating inflammatory lesions such as papules, pustules, and nodules, it does not release comedones. Although some authors suggest that cryotherapy speeds the removal of comedones and promotes acne resolution [28], the advent of effective topical remedies has largely decreased the use of cryosurgery to treat early acne lesions, especially comedones [16]. Cryotherapy is not always very effective for treating deep draining sinuses in acne [29]. According to the author’s experience in Korea, a more favorable outcome of cryotherapy tends to be obtained in patients with papulopustular or superficial cystic acne than in patients with comedones, nodules, or deep-seated cysts (Figure 4).

**Table 1:** Summary table of the results of clinical studies.

Author, Year	n	Types of Acne	Refrigerant	Delivery Method	Control	Findings
Dobes and Keil, 1940 [8]	115	Papulopustular Nodulocystic Acne scars	Solid carbon dioxide slush	Contact method		Successful results in 70% of papulopustular acne patients Hard nodules respond better than soft cysts Unsatisfactory results in acne scars
Goette, 1973 [13]	25	Comedopapular Papulopustular	Liquid nitrogen	Cotton tip	Topical medications	Cryotherapy-treated pustular acne lesions involuted more rapidly than lesions treated by topical medications
Leyden et al., 1974 [18]	25	Nodulocystic	Carbon dioxide Nitrous oxide Freon	Cryoprobe		Marked flattening or complete resolution of nodulo-cystic lesions in 7–10 days
Layton et al., 1994 [25]	11	Acne keloids	Liquid nitrogen	Spray	Intralesional triamcinolone injection	Cryosurgery proved a significantly better treatment for these more vascular keloids than triamcinolone
Röhrs et al., 1997 [24]	16	Acne keloids	Nitrous oxide Liquid nitrogen	Contact method		Excellent and good results with flattening of the lesions to the skin level or slightly persisting hypertrophy were obtained in 73% of the lesions

### 3.4. Acne-Induced Hyperpigmentation

Melanocytes are highly susceptible to freezing at temperatures of −4 to −7 °C [30]. Clinically hypopigmentation is one of the most common side effects of cryotherapy [16,26,31,32], which is characterized by an absence of melanosomes in keratinocytes, although melanocytes are preserved [33]. Recently, researchers demonstrated that the pigment-reducing properties of cooling could apply to treat benign skin pigmentation, with a high rate of procedural success and aesthetic improvement at two months post-treatment [34]. In the study, controlled, localized freezing to the epidermis was associated with the least procedural discomfort, social downtime, and minimal risk of postinflammatory hyperpigmentation. This device (Glacial Rx; R2 Technologies, San Ramon, CA, USA) is now FDA-cleared for removing benign skin lesions. A recent study by Kwack et al. [30] further evaluated the effect of a novel temperature-adjustable cryotherapy device on the expression of pigmentation-related biomarkers and found that the expression of tyrosinase, c-kit, melanocortin 1 receptor, and microphthalmia-associated transcription factor showed decreased tendency after local skin cooling under various conditions (−5 °C to 10 °C, for 5, 10, or 20 s). In patients with dark skin, acne shows a tendency to be accompanied by postinflammatory hyperpigmentation [35]. Since acne-induced pigmentation is at least as concerning as the acne lesions themselves and, in many cases, is considered even more troublesome than the acne [35], the use of cryotherapy to treat both acne and postacne hyperpigmentation is worth further investigation.

Brody [28] suggested that cryotherapy is a superlative agent for neurotic excoriations or “acne excoriée,” characterized by disfiguring hyperpigmentation of picked lesions. An earlier report by Dobes and Keil [8] also describes that neurotic excoriation of acne lesions can be successfully treated by cryotherapy. A moderate application to excoriated areas will keep the patient’s hands off the lesions long enough to heal and inspire the patient not to touch the areas for a variable length of time, with predictable results [28]. There is a report of the successful use of cryotherapy in patients with therapy-resistant prurigo nodularis [36], a neurotic excoriation that shares many features with acne excoriée. Dermatologists found that temperature-controlled cryotherapy effectively blocks itchy sensations [37].

### 3.5. Hidradenitis Suppurativa

Although hidradenitis suppurativa and acne show little overlap in pathogenesis and clinical features, they often occur together as part of the acne tetrad. They are both characterized by innate inflammation involving interleukin-1 [38]. Bong et al. [39] reported outcomes for ten patients with persistent painful nodules of hidradenitis suppurativa treated with cryotherapy. Cryotherapy was effective in eight of ten patients with limited but persistent painful nodules of hidradenitis suppurativa and was described as a possible treatment for hidradenitis suppurativa [39]. The authors postulated that post-treatment ulceration and healing by secondary intention destroy hair follicles and apocrine glands, which are involved in the pathogenesis of chronic nodule formation [39]. There has been a case report [40] of a modified intralesional cryotherapy technique called “cryoinsufflation” by using an ordinary needle to inject liquid nitrogen directly into the sinus tracts of hidradenitis suppurativa. Cryoinsufflation can also be utilized when planning surgical procedures of hidradenitis suppurativa, including deroofing and limited and radical excision [41].

### 3.6. Other Inflammatory Dermatoses

The role of cryotherapy in treating other inflammatory skin conditions is worth mentioning since acne vulgaris is regarded as a primary inflammatory disease rather than an infectious condition. Although research on the impact of individual skin characteristics is inconsistent, positive effects on reducing inflammation and oxidative stress have been noted, supported by clinical reports of cryotherapy successfully used in several inflammatory skin conditions [9,37], including atopic dermatitis [42]. Cryotherapy was also reported to be effective in treating psoriasis [43], although many clinicians have hesitated to use cryosurgery in psoriasis since cold injury may induce an isomorphic response (the Koebner phenomenon).

## 4. Mechanism of Action

### 4.1. Histologic Studies

Traditionally, freezing-related direct cellular injury is thought to be responsible for the efficacy of cryotherapy in treating acne. Acute inflammation with predominant neutrophils develops 3 to 6 h after liquid nitrogen spraying; 12 to 24 h after, the lymphocytes and macrophages are recruited [44]. Although not fully founded in evidence, it is generally regarded that freezing causes inflammatory lesions to disappear faster than without treatment [15]. This effect may explain cryosurgery’s superior efficacy in treating more severe subtypes of acne lesions, i.e., cysts and keloids [31]. As shown in histological observations, cryotherapy produces cold damage to the fibrotic cyst wall, resulting in the chemotaxis of neutrophils, whose proteases will subsequently destroy the cyst wall and allow healing [9,12]. Histological studies also found that cryotherapy eventually causes a reduction in myofibroblasts and mast cells in keloidal lesions, in addition to the normalization of collagen structure and organization [45].

Another histologic finding of cryo-treated acne lesions is vascular disruption. Histologic studies of the refrigerant sprayed on the pig skin showed vascular engorgement with extravasated red blood cells at 15 and 30 s on day zero and normal healing at a subsequent biopsy on day seven [46]. Cryotherapy exhibited significantly better results than intralesional triamcinolone in a randomized study with 11 patients with multiple acne keloids, especially in early vascular lesions [25]. Cryotherapy is effective in treating cystic acne, another subtype characterized by a distinct vascular component. In some patients, pyogenic granuloma-like vascular proliferations form in areas of cystic activity, suggesting that factors unique to cystic acne may play a role in this vascular proliferation [47]. Interestingly, pyogenic granuloma itself responds well to local cryotherapy, which aligns with the findings by Chao et al. [48] that vascular-mediated injury is responsible for the therapeutic efficacy of cryotherapy in microvascular-perfused tissue.

### 4.2. Possible Mechanisms of Action

Important factors that play a role in the genesis of acne formation include hormones, inflammatory mediators, *Cutibacterium acnes*, and genetics. Testosterone and androgens cause activation and proliferation of keratinocytes, sebaceous cells, and ductal lining cells of the hair follicle, which accumulate in the pilosebaceous unit and result in the formation of pore obstruction and more sebum production. Oxygen availability within the cells can be compromised by the pressure exerted inside the pilosebaceous unit, providing ideal environmental conditions for the growth of *C. acnes* which further promotes acne formation [49].

Several suggested mechanisms of acne cryotherapy include restoration of microflora, improvement of skin microcirculation, normalization of follicular hyperkeratosis, improvement of sebum evacuation, and immunomodulation [50]. Reversal of follicular hyperkeratosis initially has been suggested, based on the findings that cryotherapy induces molecular changes resulting in a decreased expression of Ki-67 [51], an epidermal proliferation marker that is an indicator of ductal hyperproliferation during acne development [52].

A second suggested mechanism is the immunomodulatory action of cryotherapy, which has been also studied in a variety of disorders [42]. Cold has an initial inflammatory effect, but an anti-inflammatory effect becomes evident 24–48 h after freezing the lesion, and faster reabsorption of the lesion occurs [53]. Several reports showed the clinical efficacy of local cooling in the relief of inflammatory acne [7,13,18], producing better results in papulopustular acne than in nodular lesions [8]. It has been noted that cold exposure induces the release of fewer proinflammatory cytokines (IL-2, IL-6, IL-8, IL-9, and TNF-α, among many others) and more anti-inflammatory cytokines (mainly IL-10), in addition, improve humoral and cellular immunity, stimulating B lymphocytes and natural killer lymphocytes (NK cells) [42]. Local cryotherapy has been shown to decrease the level of interleukin-1β, prostaglandin-E2, and nuclear factor-κB, which are known to be elevated in inflammatory acne lesions in vivo [54]. In keloidal lesions treated with cryotherapy, CD163+ M2 macrophages and matrix metalloproteinase-9 were significantly increased, indicating that cryotherapy-recruited macrophages supply matrix metalloproteinase-9, which function in fibrotic resolution in during treatment [55].

Cellular mechanisms governing acne pathogenesis include insulin-stimulated activation of the PI3K-Akt signaling pathways along with mTOR in sebocytes, resulting in increased synthesis of proteins and lipids, cell proliferation, and inflammation [49]. Insulin has been reported to decrease in cold conditions [56], which may imply the possible role of cryotherapy in inhibiting the activation of the signal pathways during acne development.

### 4.3. Effects on the Sebaceous Gland

Given the preferential susceptibility of lipid-containing cells to cold [57], it might be feasible to hypothesize that controlled local skin cooling causes preferential injury to sebaceous glands. According to a histologic observation by Burge and Dawber [58], a light freezing injury resulted in the shrinkage and degeneration of sebaceous glands. Furthermore, the infiltration of inflammatory cells and partial destruction of sebaceous gland cells have been found 24 h after shallow cryotherapy [59].

Some studies have evaluated in vivo biological effects of cryotherapy on normal sebaceous glands. Ray Jalian et al. [60] investigated the role of controlled local skin cooling in causing preferential injury to sebaceous glands to understand its mechanism in treating acne vulgaris. They observed that cooling-induced damage led to a 20% reduction in sebum output for two weeks, with minimal collateral injury to surrounding tissues. A higher number of freeze–thaw cycles and slower thawing resulted in more significant tissue injury. In mouse ears, peak histologic damage occurred 72 h after treatment; eosinophilic necrotic plugs formed within sebaceous glands, and the number of glands was significantly reduced up to one week after treatment [60]. A significant decrease in sebum secretion has also been reported in patients receiving whole-body cryotherapy [61]. A study using coherent anti-Stokes Raman scattering microscopy has proved a gradual loss of subcellular structures in sebocytes after cold exposure [62], which is consistent with previous findings. The effect of lipid crystallization was demonstrated to have a limited role in causing cellular disruption. Instead, sebocyte loss occurred at temperatures lower than those required for lipid crystallization [62].

Reports of successful treatment of sebaceous hyperplasia using cryotherapy [63,64,65] may support the concept of “selective cryolysis of sebaceous glands”. A recent study involving 40 patients with 517 sebaceous hyperplasia lesions found that six liquid nitrogen cryotherapy sessions at two week intervals resulted in an excellent response [76–100%] in 65.9% of patients, with no recurrence seen at the four month follow-up [66]. The authors concluded that a well-aimed and controlled use of cryosurgery is a cost-effective treatment modality for treating significant cosmetic disfigurement in patients with sebaceous hyperplasia [66]. However, the efficacy of cryotherapy for sebaceous hyperplasia seems to be inferior to electrodessication [65].

Nonetheless, the biological effect of cryotherapy on the sebaceous glands seems to be limited and reversible. Results from a study using a temperature higher than what is used in tumor treatment showed that the sebum output recovered after four weeks [60]. The study further showed that immunohistochemistry-based expression of a proliferative marker (Ki67) and a progenitor basal cell marker (keratin 15) was not disrupted by cooling, which led to the conclusion that cooling-induced damage may be temporary and may occur due to the disruption of cellular architecture, enzymatic activity, and decreased lipid content [60]. Although recovery of most sebaceous glands started 1–2 weeks after treatment, some of the glands never recovered within the experiment, suggesting that some sebaceous glands may be permanently damaged by cooling when the temperature is significantly low [32].

## 5. Adjunctive Uses of Cryotherapy in Acne Treatment

### 5.1. Cryotherapy Combined with Intralesional Injections

An alternative to monotherapy with cryotherapy in acne is to use it as an adjuvant treatment. In dermatology practice, cryotherapy has often been combined with intralesional injection therapies [36,67,68,69]. However, the efficacy of combined cryotherapy and intralesional injections has not been evaluated for acne treatment. Since keloids respond well to combined cryotherapy and intralesional injections, we may assume that keloidal acne could also benefit from combination therapy.

**Figure 5:**
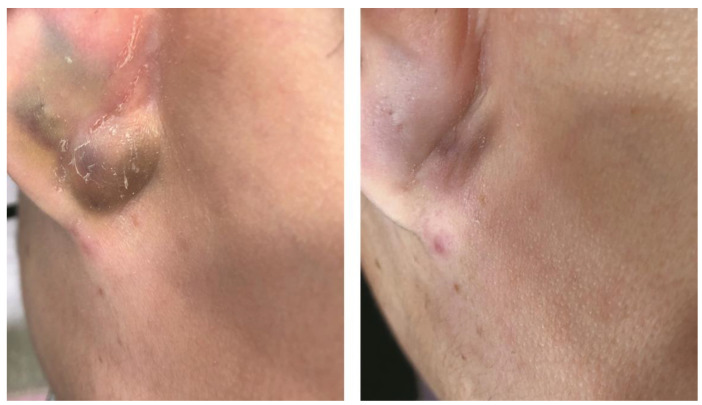
The combined cryotherapy (target temperature at −5 °C, 15 s of spraying) and triamcinolone injection (4.0 mg/mL, 0.2 mL injected intralesionally) yielded a resolution of cystic acne in a 17 year-old Korean male. Before (**left**) and two weeks after combination treatment (**right**). For cryotherapy, a temperature-controlled carbon dioxide spray device (TargetCool; RecensMedical, Ulsan, Korea) was used. Permission and consent were given for the use of the photographs.

Reports demonstrate that hypertrophic scars and keloids respond rather well to a combination of cryotherapy and intralesional corticosteroid injections. Combined cryotherapy and intralesional steroid injection are often used for small, newly formed acne keloids [70]. Some authors [71] claim that the best treatments for acne keloids are steroid injections plus cryotherapy. Optimal results, with an average of 80% improvement, have been reported with cryosurgery combined with intralesional steroid injection for early acne keloid lesions [15]. Yosipovitch et al. [72] performed a controlled study to evaluate the combined effects of intralesional corticosteroid injections with cryotherapy vs. intralesional corticosteroids or cryotherapy alone and found that the combination treatment was superior to each monotherapy. Therapeutic results may be improved, and scar recurrence reduced when intralesional corticosteroids are combined with cryotherapy [73]. Lesions treated with combination therapy require fewer procedures and have lower recurrence rates [26]. Moreover, it can produce marked flattening of most acne lesions within 48 to 72 h [74,75]. According to a recent survey of 100 dermatology healthcare professionals, the most treated lesions were cysts, followed by inflammatory papules, and pustules [76]. Many dermatologists use the combination because it is inexpensive, quick, simple, and has few side effects [77]. Figure 5 shows an example of the clinical result of combined cryotherapy and triamcinolone injection on cystic acne.

### 5.2. Intralesional Injections in the Treatment of Acne: The Pain Issue

One rationale for the adjuvant use of cryotherapy is that it could effectively reduce the pain of intralesional injection. Injection-related pain is of particular concern when treating pediatric patients, which largely limits the use of intralesional injection in children and adolescents. Pain during injection has not been a major topic in pediatric dermatology, although injections with triamcinolone has been recommended for the fast resolution of nodulocystic lesions in adolescent acne patients [75,78]. Even minor medical procedures such as venipuncture cause significant pain in the pediatric population, compounded by needle phobia in children and adolescents [79]. Nevertheless, interventions to reduce pain and distress are infrequently used [80]. About 3–4% of the world population is estimated to have severe blood injury and injection phobia [81]. Injection phobia was the main reason for 11.5% of the COVID-19 vaccine-hesitant people in the UK [82].

Dermatologists have been using local anesthetics mixed with the suspension of corticosteroids to reduce injection pain. However, although the injectable is prepared with a local anesthetic, patients receiving an intralesional injection with anesthetic experienced no decrease in pain at the time of injection, as demonstrated in a recent double-blind, randomized controlled trial [83]. Several reasons lie behind this phenomenon. First, commonly used local anesthetics, such as lidocaine and bupivacaine, can cause a burning sensation and be a source of significant discomfort due to their acidic pH [84]. Second, intralesional injections may prove challenging because of the high pressures required to deposit an adequate amount of corticosteroid [85], which could result in considerable pain associated with a rapid increase of the pressure inside the lesion. Most of all, mixing local anesthetics with corticosteroids does not diminish the pain associated with the needle puncture itself, the most noxious component of the injection experience [83,86], since the anesthetic effect takes place a few seconds after injection. A recent controlled trial by Zakria et al. [83] counterintuitively revealed that there is even more injection-associated pain when lidocaine with epinephrine is included with the corticosteroid. Based on their findings, the authors recommended diluting the intralesional injection drugs with normal saline rather than lidocaine.

### 5.3. Local Skin Cooling to Reduce Pain during Intralesional Injection

Pain with intralesional injections is considered a necessary evil, but it can be reduced by simple means, including cooling. Local skin cooling before the procedure can help minimize the pain during intralesional injection [87,88]. Cutaneous cooling for pain relief (cryoanalgesia) has a long history. Its recorded use in surgery was as early as 1807 [88], implying that it could also reduce pain during intralesional injections. Early in 1850, Arnott [2] suggested that the numbing effects of the cold should be further utilized to anesthetize skin before surgery. One of the simplest methods involves cooling the skin using ice packs held in place to be injected, which should be applied for several minutes before injection to get an effective anesthetic effect [88,89]. To achieve a skin surface temperature of 10 °C, the ice pack needs to be applied for about 20 min [90]. The time-consuming nature and the relative lack of good anesthetic effects of ice compression prompted clinicians to use other cryotherapy modalities to get more anesthetic and analgesic effects [91,92]. A recent prospective, open trial involving 21 dermatologic patients who received intralesional triamcinolone injections revealed that the mean pain reduction was 3.4 (a numeric rating scale, 0 to 10) in the liquid nitrogen spray cooling group and 6.9 in the noncooling control group (*p* < 0.001) [92].

While providing a better anesthetic effect than simple ice compression, “surgical cryotherapy” can cause significant cold injury to the skin when used incorrectly [93]. To avoid unwanted side effects such as erythema or blistering, physicians should keep a proper distance and maintain appropriate spray strength and duration [92], making the procedure hard to standardize. Dichlorotetrafluoroethane has also been used to control postsurgical pain and to decrease the pain of injections [94]. One study reported that the anesthetic effects of skin cooling using ethyl chloride spray were comparable to those of a 45 min application of topical lidocaine–prilocaine cream for pain relief during forehead botulinum toxin injections [95]. The literature review found that more research needs to be completed on whether cooling the acne lesion before intralesional injection decreases injection pain.

When treating nodulocystic acne, the author uses a cryotherapy parameter as follows: treatment temperature at −3 °C and spraying time of 9 to 12 s on each lesion. Cryospraying is performed immediately before and during intralesional triamcinolone injections. In practice, the author uses a temperature-controlled cryotherapy unit (TargetCool; RecensMedical, Ulsan, Korea), which uses pressured carbon dioxide as a refrigerant. This precision cryotherapy device enables users to keep the skin surface temperature constant during spraying with the help of a built-in real-time skin temperature monitoring system.

## 6. Side Effects

### 6.1. Pain and Discomfort

Although cooling can reduce the pain sensation, it can also induce pain and discomfort [89]. Cryotherapy is only mildly painful for a brief amount of time. Transient tingling sensation or sensory alteration can develop on the treatment site [66,96,97]. Shortly after freezing erythema and edema are likely to be accompanied by peeling, and this can be unappealing to some patients [31]. In a recent hospital-based study [21], postcryotherapy erythema was seen in 8% of patients. Cryosurgery may cause the temporary crusting of the treated cysts as an undesirable side effect [16].

### 6.2. Pigmentary Alterations

The most common side effect of cryotherapy is a pigmentary disturbance. Although hyperpigmentation can occur in some patients [26], hypopigmentation is more commonly seen after cryotherapy [30]. Burge et al. [33] have investigated the changes in pigmentation and melanocyte distribution in human skin after a standardized freeze injury. All lesions treated with 5 or 15 s of liquid nitrogen spraying developed hypopigmentation with a peripheral rim of hyperpigmentation. Abnormalities in pigmentation persisted for at least 6 months. The mechanisms underlying melanocyte’s sensitivity to cold remain unclear, but some studies have suggested that keratinocytes remain intact while melanocytes undergo different levels of apoptosis at temperatures of −4 to −20 °C for 4 min [32]. On the other hand, a histologic evaluation revealed that liquid nitrogen spraying (5 or 15 s) induced hypopigmentation, which was related to an absence of melanosomes in keratinocytes, although melanocytes were present [33]. These findings suggest that postcryosurgery hypopigmentation is not always synonymous with an absence of melanocytes. A recent study demonstrated that contact cooling of the skin surface causes selective killing and loss of epidermal melanocytes when the tissue temperature is −7.5 °C or cooler, leading to depigmentation [98], and implying that melanocyte damage may be related to the degree of cooling.

After cold-induced skin depigmentation, repigmentation occurs as melanocytes gradually migrate from hair follicles [98]. Although pigmentation changes usually recover within months [96], prolonged freezing for longer than 30 s may result in permanent pigment loss [33]. The risk for pigment abnormalities is related to the duration of freezing [33] and the number of sessions completed [26]. The risk of cryotherapy-induced hypopigmentation should always be considered in dark-skinned patients [16,31]. Cryotherapy for keloids is known to cause hypopigmentation in patients of darker skin types, the same population with a high prevalence of keloidal acne [85].

### 6.3. Scarring

Scarring is rare after cryotherapy at temperatures of −7.5 °C or warmer [98]. However, aggressive cryotherapy can cause hypertrophic or even atrophic scarring [22,43]. Since atrophic scarring is a long-term consequence of acne vulgaris that can heavily impact the patient’s quality of life, aggressive freezing may not be adequate for treating acne.

### 6.4. Rare Side Effects and Contraindications

Rarely, headaches and syncope can occur after cryotherapy in the forehead or temple area [66]. Cryogen insufflations can occur when spraying after draining the cystic lesion [53]. Cold-induced urticaria can be seen after cryotherapy [89]. Furthermore, physicians should be cautious if a patient has an underlying connective tissue disease, particularly cryoglobulinemia or cryofibrinogenemia [99].

## 7. Acne Cryotherapy: What Is the Optimal Temperature?

### 7.1. Therapeutic Temperature Range for Acne

Until now, the most effective and safe temperature and time ranges have not been elucidated when treating acne using cryotherapy, suggesting the need for further investigations. Textbooks say that superficial spraying of liquid nitrogen for 5–7 s for acne treatment is usually enough [53]. A relatively “weak” cooling might be more suitable for the treatment of papules and pustules of acne when we consider the histological findings that a light freezing injury results in changes confined to the hair follicles, whereas a “tumor dose” cryotherapy may destroy hair follicles and result in the necrosis of surrounding tissue [58,59]. In contrast, more aggressive freezing is required when treating more severe acne lesions, such as nodules, cysts, and keloids, since the incomplete resolution of fibrotic nodules or cystic walls may cause worsening of the lesion and may subsequently require surgical treatment [20].

Deep-freezing temperatures of conventional liquid nitrogen cryotherapy [9] are also not required when the cooling aims to reduce the injection pain during acne treatment. A recent study on patients receiving intravitreal injections showed that effective ocular anesthesia could be achieved using a temperature-controlled cryotherapy device at a temperature of −15 °C [100]. Considering that the cooling rate in the skin layer is much higher than in the vitreous body of the eyeball, the use of an even more ambient temperature is usually enough for skin anesthesia. A weak cooling of around −3 °C is sufficient in reducing injection pain, as shown in a study by Jung et al. [97].

One of the proposed mechanisms of action showing how cryoanalgesia works are that the lowered temperature slows the neuronal metabolic rate and the speed of neuronal conduction [101]. In this regard, cryosurgery-grade, extremely low temperature is too aggressive when used for pain reduction. The “counterirritant” effect, which occurs when a temperature stimulus overrides a painful stimulus in the same area and causes a reduction in the perception of the painful stimuli [94], may also involve “cooling” rather than “freezing”. In contrast to deep freezing, relatively warmer cryotherapy temperatures do not result in permanent nerve damage and cause only temporary blocks of neuronal conduction [102]. The antipruritic effect of local cooling to warmer than the temperature of −5 °C is regarded to be related to the modulation of the transient receptor potential cation channel subfamily M member 8 (TRPM8) and TRPM8- expressing sensory neurons [37], which is also a target of pain reduction [103].

Recently, Leal-Silva et al. [104] performed a controlled clinical trial to evaluate the safety and efficacy of cryotherapy as a potential treatment for inflammatory acne. In ten patients with inflammatory acne, one side of the face was treated for a maximum period of 5 min at −15 °C, and the other was used as a control. Two treatments, with a four week interval between them, were performed. Incremental significant reductions of the inflammatory lesions of acne were measured in all the subjects at one week and one month after the first treatment, and an even better improvement was recorded one month after the second treatment. Although the number of lesions increased by the third month follow-up visit, all subjects exhibited fewer inflammatory lesions than at the beginning of the study. No significant adverse events were observed. The result of the study imply that a nondestructive temperature setting can be effectively used for acne cryotherapy. This is an important finding since traditional liquid nitrogen cryotherapy has limitations in terms of its safety profile when treating acne.

### 7.2. Monitoring and Control of Target Temperature during Cryotherapy

With a temperature of around −10 °C, cooling damages sebaceous glands, disrupts some enzymatic activities, and reduces sebum output for two weeks, with minimal injury to surrounding tissues [60]. These findings suggest that treatment of sebaceous gland disorders may be achievable through brief, noninvasive skin cooling, implying less aggressive cryotherapy as an effective treatment of acne vulgaris using controlled cooling. Unfortunately, conventional cryotherapy has been used at a fixed temperature for each cryogen under various time conditions and freeze–thaw cycles [30,105]. Since the traditional cryotherapy technique is empirical and largely dependent upon the physician’s experience, it is very hard to standardize the clinical practice. The proper technique, involving proper distance from the skin surface, spraying time, and controlling the white frosting degree of the skin [93], is crucial to use conventional liquid nitrogen cryotherapy safely. As yet, a consensus on the parameters of liquid nitrogen cryotherapy for acne treatment has not been reached.

**Figure 6:**
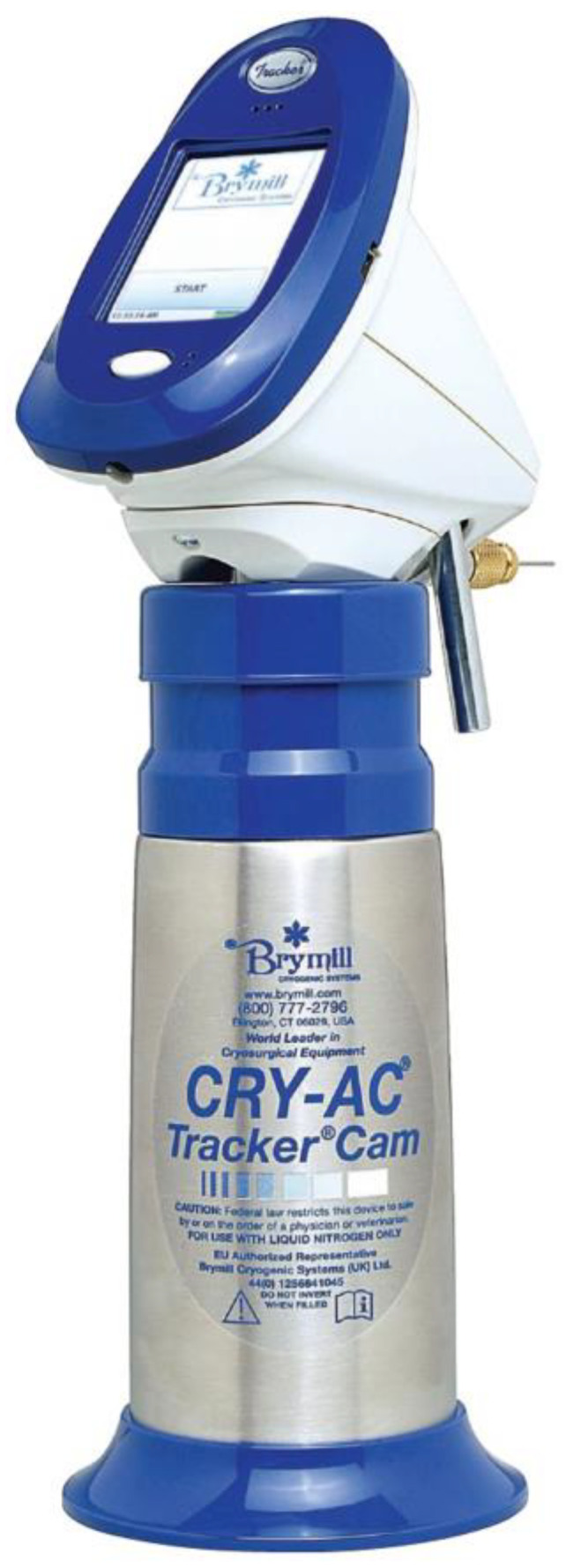
An updated cryotherapy device (Cry-Ac Tracker Cam) that measures the skin temperature when spraying liquid nitrogen on the selected skin lesion using an infrared sensor. The operator can set the device to indicate when a predetermined freeze temperature has been achieved at the lesion (courtesy of Brymill Cryogenic Systems, Ellington, CT).

One of the important technological innovations in dermatologic cryosurgery is the incorporation of the temperature sensor unit in the device (Cry-Ac Tracker Cam; Brymill Cryogenic Systems, Ellington, CT; Figure 6). Using a real-time temperature monitoring, this liquid nitrogen cryotherapy device facilitated the controlled timely destruction of the target tissue [106]. The infrared light sensor continuously and safely monitors skin temperature, along with the indicators showing how fast skin temperature decreases [107], while still lacking a free adjustment of the desired temperature.

**Figure 7:**
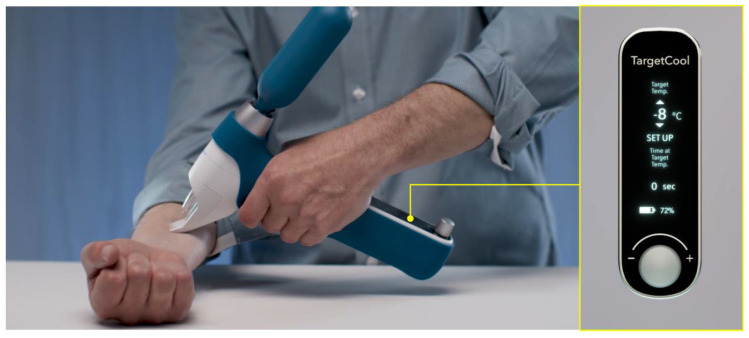
A novel cryotherapy device (TargetCool) developed to provide precise, controlled tissue cooling to reduce pain and inflammation of local procedures. This device uses pressurized carbon dioxide as a cryogen and can actively monitor and maintain the target temperature at a preset temperature (courtesy of RecensMedical, Ulsan, Korea).

The limited ability to adjust target temperature highlighted the need for accurate control of the target temperature during cooling and prompted the development of a “precision cryotherapy” device. Precise control of target temperature is based on an enhanced understanding of nozzle design and cryogen spray characteristics, such as mean droplet size, velocity, temperature, evaporation rates, and their relation to the heat extraction rate from the skin surface [108]. An example of such a precision cryotherapy device (TargetCool; Figure 7) features a unique capability of regulating the thermodynamic state (temperature and pressure) of cryogenic substances (e.g., carbon dioxide) by applying heat to cryogenic substances. A real-time temperature reading by an infrared sensor is used to measure the error between the set cooling temperature and the target temperature, and a feedback controller is used to calculate the heat required to achieve a desired thermodynamic state of cryogen substance, leading to rapid and precision cooling at the target area [30]. This technology has been reported to be an effective and safe modality to provide cooling anesthesia to the eye as local anesthesia for intravitreal injections [100]. It has been shown to help reduce pain during laser tattoo removal [97]. Another recent study shows the clinical efficacy of a contact-type cryolipolysis device (CoolSculpting System; ZELTIQ Aesthetics, Pleasanton, CA, USA) in treating inflammatory acne [104]. Using temperature control during the treatment, these devices are expected to be helpful in training other physicians to perform cryotherapy reliably [107].

## 8. Conclusions

After nearly two centuries, cryotherapy is still gaining interest in several fields of medicine, including dermatologic surgery. Dermatological cryotherapy has long been used to treat various types of acne, especially nodulocystic and keloidal subtypes. Additionally, it is a convenient, instant, and effective local anesthesia in acne patients anxious about intralesional injection-associated procedural pain. With the development of advanced cryotherapy devices, it is now possible to monitor and precisely control the target temperature, enabling physicians to standardize the protocol and minimize the adverse effects of local cryotherapy. Advances in technology are expected to promote acne cryotherapy and make it widely applicable. However, further controlled trials with larger sample sizes are needed to confirm the efficacy and provide the scientific basis for cryotherapy in acne treatment.

## Data Availability

Not applicable.
